# The dose distribution of medium energy electron boosts to the laryngectomy stoma

**DOI:** 10.1120/jacmp.v2i1.2626

**Published:** 2001-01-01

**Authors:** Ellen D. Yorke, Alireza Kassaee, Todd Doyle, Laurie A. Loevner, David I. Rosenthal

**Affiliations:** ^1^ Memorial Sloan‐Kettering Cancer Center 1275 York Avenue New York New York 10021; ^2^ University of Pennsylvania 3400 Spruce Street Philadelphia Pennsylvania 19104

**Keywords:** electron dosimetry, air cavities, head and neck radiation therapy

## Abstract

An en face, medium energy electron boost of approximately 10 Gy is often given to stomal and peristomal tissues. Because the boost is considered a simple treatment, CT‐based treatment planning is rarely used. Further, the results of such a plan might be inaccurate, as the complex dose distribution surrounding the stoma air cavity is poorly modeled by many treatment planning systems. We constructed three phantoms—two with a central vertical cavity to mimic the size and shape of the stoma and proximal trachea and one with a cavity inclined at 45° to the horizontal to better simulate anatomy. These were used to investigate the dose distribution surrounding the stoma. In all cases, the entrance to the stoma opening was centered in a field defined by a 7‐cm circular cutout and the phantom was irradiated at a source‐surface distance (SSD) of 100 cm with either vertically incident 9‐ or 12‐MeV electrons. Film measurements were made at a range of depths below and lateral to the cavity. For the vertical cavity phantoms, diode measurements were performed and isodose plans using CT scans of the phantoms were generated on a modern treatment planning system. For these two phantoms, the combined effects of lateral scatter from surrounding material and reduced equivalent thickness for electrons which pass directly through the cavity increases the dose within a centimeter of the bottom of cavity by as much as 50% for 9 MeV and 70% for 12 MeV. In material at the shallower (“superior”) end of the inclined cavity, a 40–50% overdose was noted. The dose increase is geometry dependent and is not predicted by the available treatment planning system. The potential of such a dose increase to affect normal tissues such as the neopharynx should be considered.

PACS number(s): 87.53.–j, 87.66.–a

## INTRODUCTION

Post‐operative radiation therapy is used following total laryngectomy for cancers of the laryngopharynx when clinical and/or pathological findings suggest that stomal and peristomal tissues are at risk for local‐regional recurrence. The stoma is most often treated with a single low anterior neck photon field of energy 6 MV or less. However, the dose from this field is limited by the desire to keep the spinal cord dose to below approximately 45 Gy. If the stoma and peristomal tissues are at high risk, an additional 10–15 Gy boost is often delivered with a single anterior 9‐or 12‐MeV electron field.

**Figure 1:**
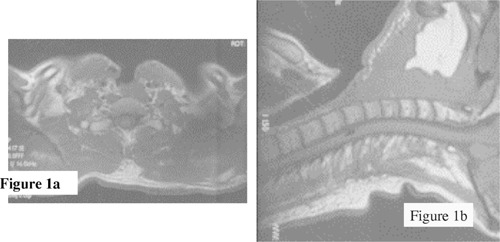
Transverse and sagittal magnetic resonance images views of a stoma and the surrounding anatomy.

Figure 1 shows the complex anatomy surrounding the stoma. On a transverse slice through its center [Fig. 1(a)], the stoma appears as a several centimeter deep air cavity in soft tissue, located at the entrance surface. In the sagittal view [Fig. 1(b)] the surgical cavity is seen to connect with the trachea, forming an air‐filled tube which runs inferior and posterior at a patient‐specific angle with the anterior surface. Despite the presence of significant tissue inhomogeneity, CT planning and advanced dose calculation methods are seldom used for electron boosts to the stoma, which are considered to be simple fields. Rather, the coefficient of equivalent thickness (CET) method^1^ ^–^ ^3^ is often employed to calculate the dose under the cavity and especially to estimate the dose to the cord. From CET calculations, one expects that the dose at a depth *d* in the soft tissue below the bottom of the cavity is approximately equal to the dose at the same depth below the surface of a flat, uniform tissue phantom.

However, lateral electron scatter produces a complex dose distribution in the soft tissue surrounding a cavity. Khan's textbook^2^ reproduces a figure^4^ which shows the dose distribution of an electron beam of unspecified energy, normally incident on a water equivalent phantom with a hole in its entrance surface. In this example, small volumes of the phantom at shallow depths directly below the cavity receive a dose which is 20–40 % higher than the maximum dose delivered by an identical beam incident on a homogeneous water phantom. Material lateral to the cavity may be underdosed. Neither the CET method nor many algorithms implemented on available treatment planning computers can correctly account for lateral electron scatter. If these high dose regions occur for the electron energies used for stomal boosts, patients treated with altered fractionation or especially with concurrent chemotherapy are at increased risk for stricture or ulcer of the neopharynx. Additionally, unexpected underdose to tissues lateral to the stoma may risk local recurrence. To better understand the implications of electron scatter in this clinical application, we measured the dose distributions of 9‐ and 12‐MeV electron beams incident on phantoms with air cavities of typical stoma dimensions.

## METHODS AND MATERIALS

Measurements were made with 9‐ and 12‐MeV electron beams because these energies are often used for stomal boosts. For all measurements, the electron beam of a Varian 2100C accelerator was vertically incident at SSD=100 cm to the top surface of a stoma phantom. In all cases, the stoma hole at the entrance surface was centered in the field defined by a 7‐cm‐diameter cerrobend cutout in the 10×10 cone.

**Figure 2:**
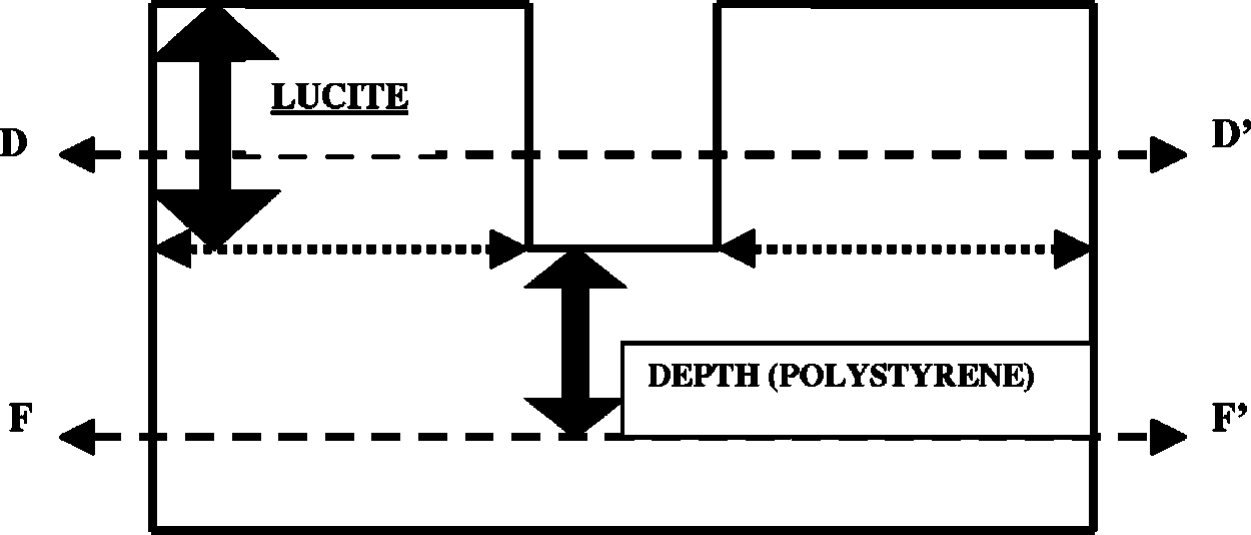
Diagram of a vertical cavity phantom which mimics stoma geometry. Lines *FF‘* and *DD‘* indicate locations where films may be placed.

We constructed three phantoms suitable for film dosimetry with Kodak “ready packs” V‐film™. Two simple phantoms employed vertical cavities, shown schematically in Fig. 2, to investigate the simpler aspects of electron dose distributions in the clinical situation. The cavity dimensions were based on averages determined from the treatment CT scans of 20 patients at the University of Pennsylvania. These showed that the cross section shape may be either circular or elliptical. An elliptical phantom was constructed by drilling identical elliptical holes at the center of two $30\times 30{\text{cm}}^{2}$ slabs of PMMA (i.e., lucite) of thickness 2.5 cm and 0.9 cm, respectively. The ellipse cross section was 2 cm×1.7 cm and its total depth was 3.4 cm. A second, circular stoma phantom was constructed by drilling a 2.5‐cm‐diameter circular hole of depth 3 cm at the center of a single 3‐cm deep slab of PMMA. These phantoms were placed on top of a stack of standard $25\times 25{\text{cm}}^{2}$ polystyrene slabs. Film could be sandwiched between polystyrene layers, as indicated by lines *FF‘* in Fig. 2. Depths below the bottom of the cavity chosen were 0, 0.1, 0.2, 0.5, 1.0, 2.0, and 4.0 cm. The deepest depth represents the cord. For the two‐layer elliptical phantom, the dose to material surrounding the cavity at 0.9 and 2.5 cm below the top surface was also investigated by sandwiching a film between layers (the dotted line *DD‘* in Fig. 2). Although this film crossed the cavity, only the dose distribution lateral to the cavity was examined. Each film was exposed to 27 MU. These conditions would deliver a dose of 27 cGy at $d_{\max}$ in homogeneous, water‐equivalent phantom, as the measured output factor of the cutout is 1.00.

**Figure 3:**
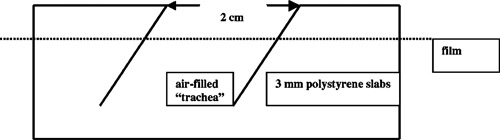
Schematic diagram of the stoma‐trachea phantom.

The third phantom was designed to investigate the effect of the inclined connecting air‐filled tracheal tube [Fig. 1(b)]. We shall refer to this as the stoma‐trachea phantom. Circular holes of approximately 2 cm in diameter were machined in a series of 0.3‐cm‐thick slabs of polystyrene (area $25\times 25{\text{cm}}^{2}$). Each hole was offset from those in the adjacent layers so that when the phantom was assembled, the holes formed an air‐filled tube descending at an angle of approximately 45° as shown schematically in Fig. 3. To aid comparison with the anatomy shown in Fig. 1(b), throughout this text, the shallow “superior” end of the phantom “trachea” is oriented to the right side of the page. “Ready‐pack” film can be sandwiched between any pair of layers. Each exposure in this phantom was at 35 MU. The known phantom geometry was used to determine where a film crossed cavity at its depth, as film exposure here would not represent dose to tissue.

Additionally, the outline of the circular hole directly above the film was lightly traced with soft pencil on the film jacket and a pinhole pattern was pricked well outside the exposed area prior to dose delivery. This pattern was used to realign the developed film with its jacket so that lines could be drawn (again, well outside the exposed area) to indicate the diameter of the “trachea” in the plane of the film.

All film dosimetry was performed with Kodak V‐film™ (10″×12″ “ready pack”). Air pockets were eliminated by smoothing the film before insertion between phantom layers, pricking pinholes at the jacket edge beyond the exposed area, and tightly compressing the film between phantom layers. Calibration curves to convert film density to dose were generated for both energies by exposing films at $d_{\max}$ in uniform polystyrene phantom (100 cm SSD, 10×10 open cone). All film for a given experiment was taken from a single package, a fresh set of calibration films were exposed, and the experimental films and associated calibration films were developed in a single run using the clinical film processor (Kodak RP X‐omat). Films were digitized with a Lumisys™ film scanner and cross beam profiles were analyzed with the program “NIH Image.”™

For the two vertical cavity phantoms, point measurements were also made with a Scanditronix™ electron diode at selected depths along the central axis under the cavity. The diode was oriented with its long axis vertical in a specially drilled polystyrene slab. Normalizing diode measurements were also made at each energy in a uniform, flat phantom on the central axis under $d_{\max}$ thickness of polystyrene for the same number of monitor units.

To investigate the predictions of a modern, commercial treatment planning system for the electron dose distribution surrounding a stoma, the two vertical cavity phantoms were scanned on a GE 9800 CT scanner and the images were downloaded to the Helax™ treatment planning system at the University of Pennsylvania. Dose distributions were generated for the same electron beams as were used for the measurements. The calculated dose distributions were normalized to 100% on the central axis at 1.0 cm below the bottom of the cavity.

## RESULTS

### Patient stoma dimensions

Measurements of the stoma region visualized on the CT scans of 20 patients found a range of cross sections, depths, and cord depths. The cavity depth is measured from the patient's anterior surface to the neopharynx. The dimensions of the elliptical phantom are the averages of these measurements. The results are summarized in Table I.

**Table I:** Stoma dimensions measured from CT scans.

	Length (cm)	Width (cm)	Cavity depth (cm)	Cord depth (cm)
Mean	1.7	2.0	3.5	7.3
Range	1.2–2.5	1.45–2.00	2.2–4.8	5.7–8.0

### Film dosimetry

**Figure 4:**
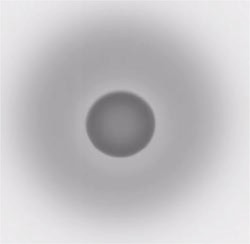
The ring pattern on a film exposed to 12‐MeV electrons at 0.2 cm below the circular vertical cavity phantom.

**Figure 5:**
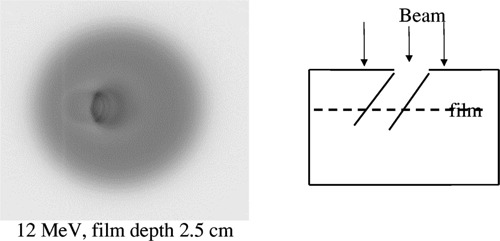
The pattern on a film exposed to 12‐MeV electrons at 2.5 cm below the surface of the stoma‐trachea phantom. The diagram shows the orientation of the film relative to the phantom.

Films exposed to 12‐MeV electrons at shallow depths beneath both vertical cavity phantoms showed a concentric ring pattern indicative of the dose distribution, as shown in Fig. 4. A more complicated pattern is seen on films exposed in the stoma‐trachea phantom. At the right side of the film, artifacts may be generated by the slight stepping of the 3‐mm slabs of which the phantom is composed. A typical pattern is shown in Fig. 5.

**Figure 6:**
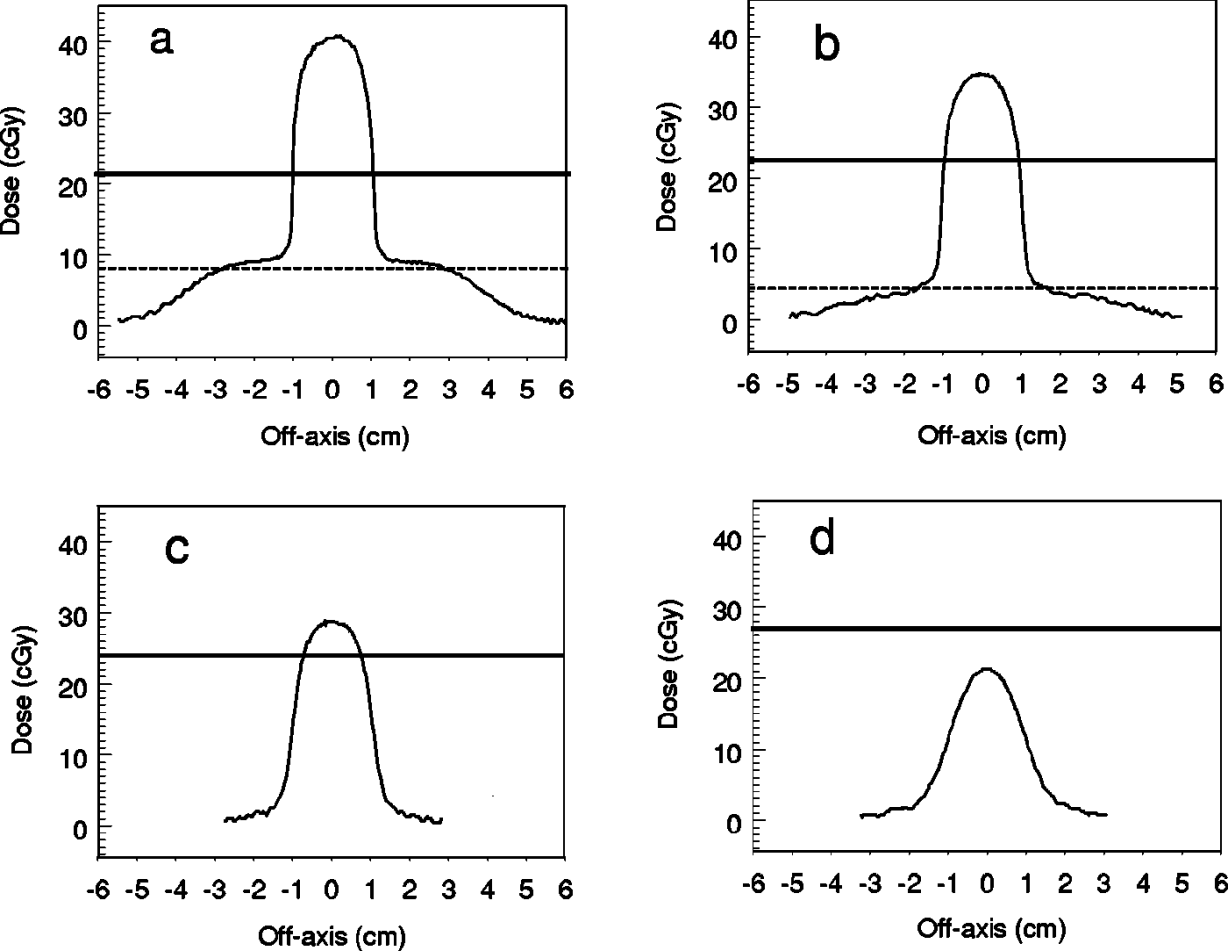
9 MeV electron dose profiles along the 2.0 cm axis of the elliptical phantom at depths below the bottom of the phantom of (a) 0.1 cm, (b) 0.5 cm, (c) 1.0 cm, and (2) 2.0 cm.

**Figure 7:**
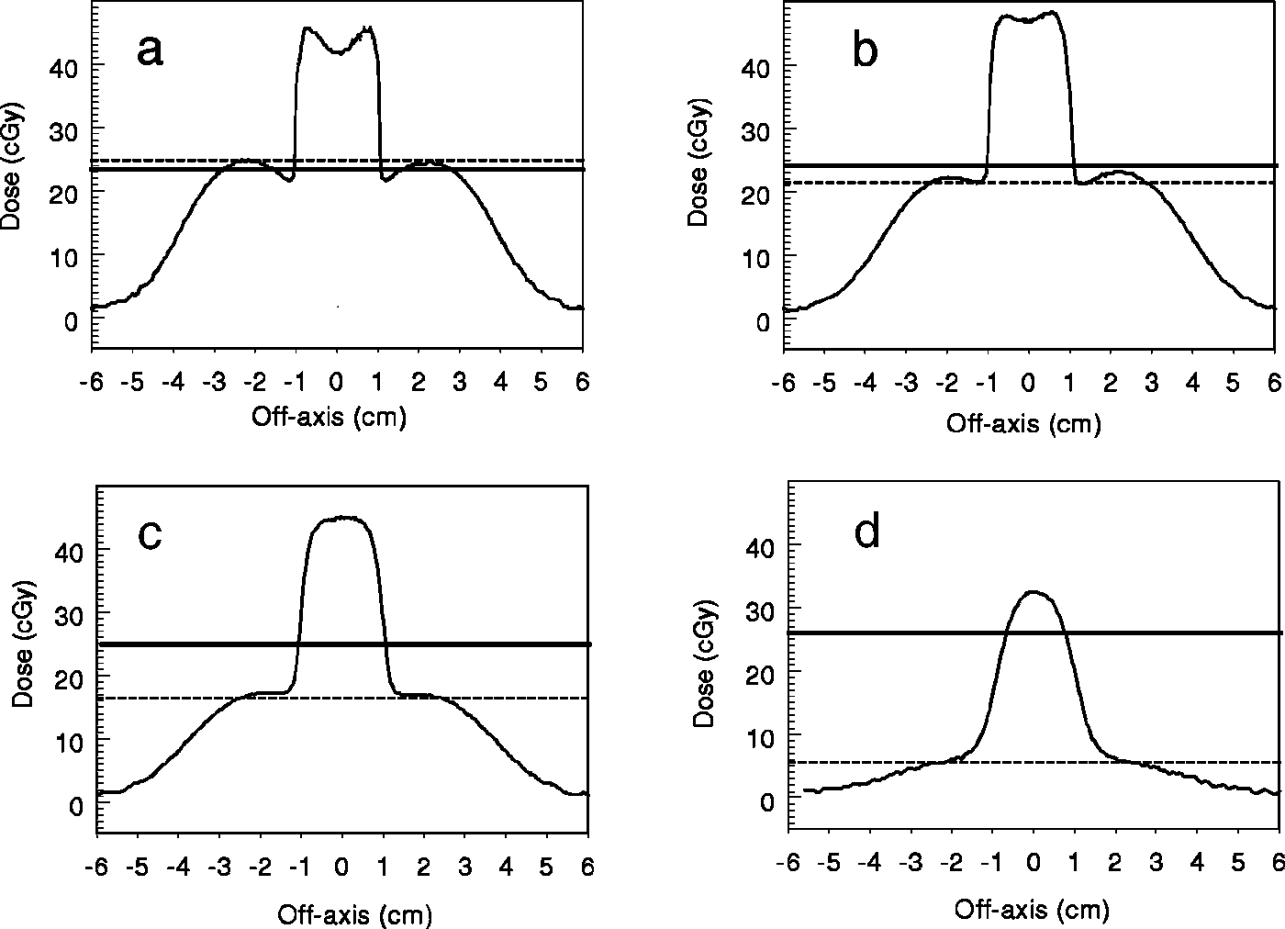
12 MeV electron dose profiles along the 2.0 cm axis of the elliptical phantom at depths below the bottom of the phantom of (a) 0.1 cm, (b) 0.5 cm, (c) 1.0 cm, and (d) 2.0 cm.

Dose profiles along the longer (2.0 cm) axis of the elliptical vertical cavity phantom at depths of 0.1 cm, 0.5 cm, 1.0 cm and 2.0 cm below the cavity are shown for the 9‐MeV beam in (Figs. 6a–6(d) and the 12‐MeV beam in Figs. 7(a)–7(d). The solid horizontal lines in these figures represent the central axis doses calculated by using the CET method together with the open field percent depth dose curves with the geometric depth equal to the depth of the film below the bottom of the cavity. The dotted line is the central axis dose calculated similarly at a geometric depth in uniform phantom equal to the distance from the top surface of the phantom to the film. This line is omitted at depths exceeding the practical electron range. Following Table VII of TG‐25 (3) the effective density of PMMA is taken as 1.115 and of polystyrene as 0.99.

A striking increase in dose compared to that calculated with the CET method is seen at shallow depths directly below the cavity—up to approximately 1.0 cm for 9 MeV and 2.0 cm for 12 MeV. Within this range, doses under the cavity exceed the $d_{\max}$ dose in flat phantom by more than 10%, with peak values exceeding by as much as 50% for 9‐MeV and 70% for 12‐MeV beams. The 12‐MeV profiles shown at 0.1 cm and 0.5 cm also have a distinct structure. This is lost by 1.0 cm depth. There are high dose “horns” near the inner edge of the shadow of the cavity, a dip adjacent to the outer edge of the cavity's shadow which is followed by a rounded, shoulderlike peak and then the dose fall‐off in the field penumbra. This structure was not seen in the 9‐MeV profiles for the elliptical cavity. These features are caused by the combined contributions of electrons which enter the phantom directly through the cavity and are still in the high part of the percent depth dose curve and electrons which enter through the PMMA surrounding the cavity and have traversed several cm of material and experienced significant lateral scatter but have not yet exceeded their range. The dose in the irradiated field lateral to the cavity shadow is similar to that predicted by the CET method (dotted line).

At 4‐cm depth directly below the cavity, the dose is lower than expected from CET calculations. For the 9‐MeV electrons, the film exposure at 4‐cm depth was almost indistinguishable from fog while CET calculations predicted a central axis dose of 6.1 cGy. For the 12‐MeV electrons, the dose distribution resembled Fig. 7(d), with a peak value of approximately 10 cGy while CET calculations predicted 24.2 cGy. By 4 cm below the cavity, electrons which entered through material surrounding the cavity have “ranged out” while the cavity provides small aperture )2.0 cm×1.7 cm) tertiary collimation for electrons which are directly incident upon it. The depth dose at 4 cm beneath the cavity is therefore similar to that of a cutout of the same areal dimensions as the cavity. The reduction in electron depth dose for small fields is well documented in the literature.^3^ ^,^ ^5^

**Figure 8:**
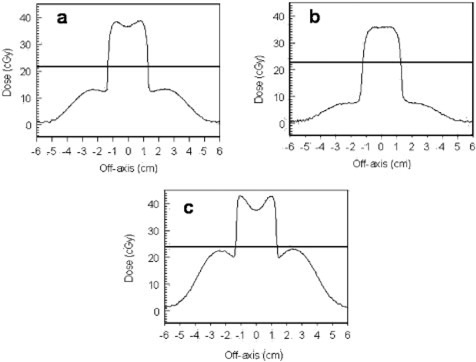
Dose profiles under the circular stoma phantom: (a) 9 MeV at depth of 0.1 cm, (b) 9 MeV at depth of 0.5 cm, and (c) 12 MeV at depth of 0.5 cm.

Figures 8(a) and 8(b) show the dose profiles at 0.1 and 0.5 cm beneath the circular vertical cavity phantom for the 9‐MeV beam. Figure 8(c) is the 12‐MeV profile at 0.5 cm. The solid horizontal line was calculated as in Figs. 6 and 7. Figure 8 shows that the dose perturbation depends on the cavity size and shape. For this wider but shallower cavity, the hornlike structure is more pronounced and is seen for the 9‐MeV electrons at 0.1 cm as well as for the 12‐MeV electrons. The dose on the central axis is lower at the depths where the horns are most prominent [Figs. 8(a) and 8(c)] and larger [8(b)] at deeper depths than for the elliptical cavity.

**Figure 9:**
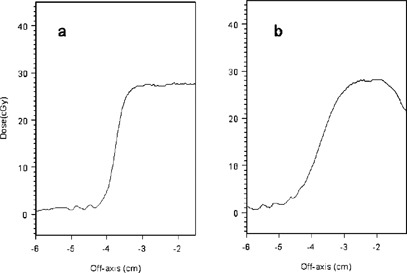
12 MeV dose profiles along the extension of the major axis of the elliptical phantom outside the cavity at depth of (a) 0.9 cm and (b) 2.5 cm.

Figures 9(a) and 9(b) are dose profiles outside the elliptical cavity for 12‐MeV electrons at 0.9 cm and 2.5 cm below the top surface of the phantom (line *DD′* in Fig. 2). At 0.9 cm, the profile is quite flat at a dose of approximately 27 cGy except for a narrow dip to 21 cGy near the cavity wall. At 2.5‐cm depth, the profile dips to approximately 21 cGy near the cavity wall and then rises to approximately 27 cGy at the top of a rounded “shoulder” before falling off in the penumbra.

The doses expected from CET calculations at these two depths are approximately 24.8 and 27 cGy, respectively. For the 9‐MeV electrons, the profile shapes at the two depths are similar to those shown for 12 MeV except that the maximum dose is 24 cGy and the minimum near the wall is 18 cGy. The doses expected from CET calculations are approximately 24.2 cGy at 0.9‐cm depth and 23.6 cGy at 2.5 cm.

**Figure 10:**
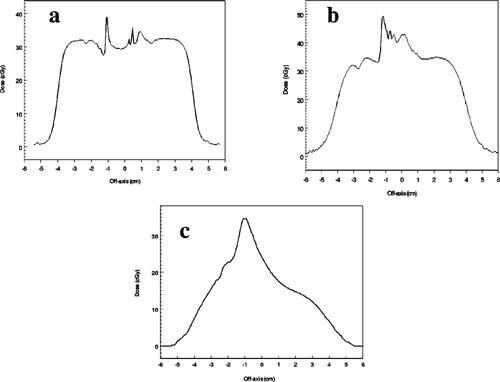
Profiles from films exposed to 12‐MeV electrons in the stomatrachea phantom at depths of (a) 1.5 cm, (b) 2.4 cm, and (c) 3.9 cm. The $d_{\max}$ dose in uniform water‐equivalent phantom would be 35 cGy.

**Figure 11:**
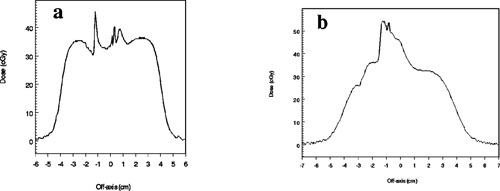
Profiles from films exposed to 9‐MeV electrons at depths of (a) 1.5 cm and (b) 2.4 cm. The $d_{\max}$ dose in uniform water‐equivalent phantom would be 35 cGy.

Figures 10 and 11 are dose profiles measured in the stoma‐trachea phantom. Figures 10(a)–10(c) show 12‐MeV profiles along the horizontal line bisecting a film similar to that of Fig. 5 at depths of 1.5, 2.4, and 3.9 cm below the surface of the phantom. Figures 11(a) and 11(b) are similar profiles for 9‐MeV electrons at depths of 1.5 and 2.4 cm. In each graph, the central axis passes through x=0. The tracheal tube slopes so that it deepens as *x* decreases [see Figs. 1(b), 3, and 5]—relative to patient anatomy, *x* decreases as one moves inferiorly. The scalloping irregularities on the right side of each graph are artifacts of the phantom construction from 0.3‐cm polystyrene slabs. Results for the simpler vertical cavities are helpful in understanding the main features of the profiles.

In Fig. 10(a), the high dose peak is on the air side of the “trachea”‐polystyrene interface, and would not be clinically significant. Except for the artifacts, the dose in the polystyrene surrounding the cavity is close to that expected in uniform phantom, similar to the situation outside the vertical cavity (Fig. 9). At 2.4‐cm depth, the high dose peak is in polystyrene, suggesting that a small volume of soft tissue at the superior side of the trachea may receive 40% higher dose than $d_{\max}$. The location of this material is similar to that near the “horns” at shallow depths below the vertical cavities. High dose results from combined contributions of electrons which have scattered while passing through the deeper polystyrene “superior” to the cavity and electrons which have passed directly through the cavity. A larger volume of soft tissue in the shadow of the cavity at this depth receives an approximate 20% overdose. At 3.9‐cm depth the peak is due to electrons which pass through air followed by approximately $d_{\max}$ of polystyrene. However, unlike what is observed for the vertical phantoms, the overdose at 9 MeV (Fig. 11) is slightly greater than for 12 MeV.

### Diode measurements in vertical cavity phantoms

**Figure 12:**
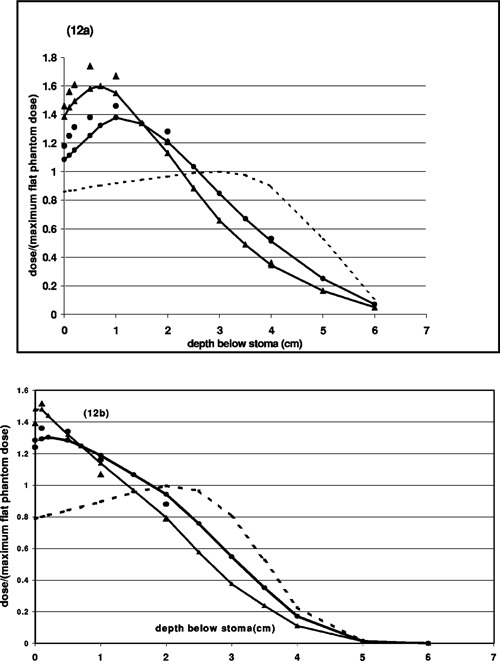
Central axis doses below cavity, normalized to $d_{\max}$ in uniform phantom. Circles and triangles represent measurements for the circular and elliptical vertical phantom respectively. Diode measurements are connected by solid lines. Film measurements are discrete symbols. Dotted lines are the percent depth dose curves from clinical data tables.

The central axis diode measurements beneath the two vertical cavity phantoms are in qualitative but not strict agreement with the film dosimetry. They agree within 5% for 9‐MeV and within 15% for the 12‐MeV electrons. Nonetheless, the diode measurements confirm that there is a large dose increase beneath the cavity. Figure 12 compares film measurements, diode measurements and CET calculations of the central axis dose at specified depths below the bottom of the cavity for 12 MeV [Fig. 12(a)] and 9 MeV [Fig. 12(b)] electrons. Each curve is normalized to the measured dose at $d_{\max}$ in uniform phantom. The circles are data for the circular cavity, the triangles for the elliptical cavity. The dotted lines are the calculated uniform phantom depth dose curves.

### Treatment Planning System Comparison

**Figure 13:**
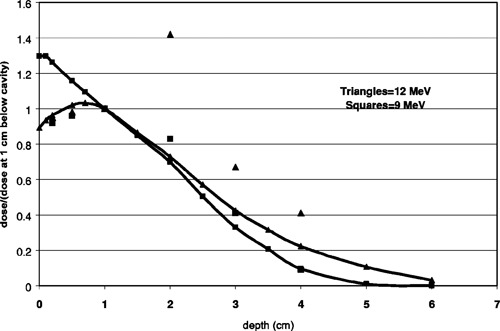
Relative dose along the central axis beneath the vertical elliptical phantom as measured with the diode (connected curves) and calculated by the treatment planning system (discrete symbols). Doses are normalized to 1.0 cm below the bottom of the cavity.

Figure 13 compares the treatment planning system calculations on central axis for the elliptical phantom with the diode readings, both normalized at 1 cm below the bottom of the cavity. For 9 MeV (squares) the agreement is good. For 12 MeV (triangles), the treatment planning system predicts a high dose region approximately 2 cm below the cavity, contrary to measurements. For both energies, the calculated isodose distributions predict that material lateral to the cavity at 1–2 cm below the surface of the phantom receives approximately the same dose as the normalization point. This is contrary to the film measurements, which showed that the dose lateral to the cavity is approximately equal to the expected dose in flat phantom, i.e. approximately half of the central axis dose at 1.0 cm below the cavity.

## DISCUSSION

9‐ and 12‐MeV electrons are often used to boost stomal and peristomal tissues. Our measurements suggest that, for these energies, some soft tissue surrounding the cavity is significantly higher than the $d_{\max}$ dose in homogeneous tissue delivered by the same beam. The increase, which is due to lateral scattering, depends strongly on the cavity geometry. For vertical cavities of typical stoma dimensions, the increase is greater and extends to deeper depths for 12‐MeV than for 9‐MeV electrons. For 12‐MeV electrons, the dose increase may exceed 150% for tissue within 1 cm of the bottom of the cavity. For 9 MeV, the increase is easily greater than 130% and the maximum increase may be close to 150% within 0.5 cm of the cavity base which, in a patient, would include tissue of the neopharynx. A substantial (40%) dose increase is also observed in tissue‐equivalent material near the shallow (superior) end of a cavity inclined at approximately 45°, which better models the connection between trachea and stoma.

Therefore, small volumes of the soft tissue of the neopharynx receive electron boost doses which are 30% to 50% larger than anticipated from homogeneous tissue or CET method estimates. For a boost prescription dose of 10 Gy, this amounts to 3 to 5 Gy more than expected, delivered at higher than expected dose per fraction. Since electron stomal boosts are common and most patients do not experience soft tissue complications, the average tolerance dose of these tissues to small “hot” volumes may be high. Nonetheless, the potential of the high dose regions for producing soft tissue complications leading to stricture or ulcer of the neopharynx should be considered when deciding between 12‐ and 9‐MeV electrons for an individual patient's stomal boost.

The dip in the dose profile at the outer edge of the vertical cavity shown in Fig. 9 indicates that small volumes surrounding the cavity receive approximately 20% lower dose than expected from uniform medium calculations. The underdose is more severe for 9 MeV. Potential underdose may be considered when choosing the electron energy if tissue surrounding the cavity is at high risk for tumor clonogen infestation. However, no such effect was seen at shallow depths surrounding the stoma‐trachea phantom. At worst, the volumes involved are small and the entire region has already received more than 45 Gy from the anterior neck photon field, especially if the photon prescription depth was 3 cm rather than $d_{\max}$.

Our measurements at 4 cm below the bottom of the vertical cavities indicate that the CET method provides a conservative estimate of the cord dose. The spinal cord is unlikely to be a problem because of its depth and the additional density provided by bone. However, because of the serious nature of transverse myelitis and the interpatient variability in stoma and cord geometry summarized in Table I, we believe it is reasonable to make a CET method estimate of the cord dose based on an individual patient's anatomy, especially when considering a 12‐MeV electron boost. A lateral simulator film is sufficient to localize the cord depth.

Although the simplistic nature of the CET method is acknowledged (3), CT‐based treatment planning with advanced dose calculation algorithms is usually not performed for the electron stomal boost. Even in exceptional clinical situations, such planning would only be warranted if the treatment planning system's dose calculation algorithm provides accurate information in this complex scattering geometry. One modern commercial system available to us reproduces some, but not all, of the features observed with measurements. There is a recently published report of another commercial system which deals accurately with electron lateral scatter in severely inhomogeneous geometries^6^ and the future is likely to produce more. In the absence of such a system, measurements should be considered when clinically relevant.
